# Translational control of enzyme scavenger expression with toxin-induced micro RNA switches

**DOI:** 10.1038/s41598-021-81679-6

**Published:** 2021-01-28

**Authors:** Nina M. Pollak, Justin J. Cooper-White, Joanne Macdonald

**Affiliations:** 1 Genecology Research Centre, University of the Sunshine Coast, Sippy Downs, QLD Australia; 2 School of Science, Technology, and Engineering, University of the Sunshine Coast, Sippy Downs, QLD Australia; 3 Australian Institute for Bioengineering and Nanotechnology, The University of Queensland, Brisbane, QLD Australia; 4 CSIRO Synthetic Biology Future Science Platform, GPO Box 1700, Canberra, ACT Australia; 5 UQ Centre for Stem Cell Ageing and Regenerative Engineering, The University of Queensland, Brisbane, QLD Australia; 6 Division of Experimental Therapeutics, Department of Medicine, Columbia University, New York, NY USA

**Keywords:** Biochemistry, RNA

## Abstract

Biological computation requires in vivo control of molecular behavior to progress development of autonomous devices. miRNA switches represent excellent, easily engineerable synthetic biology tools to achieve user-defined gene regulation. Here we present the construction of a synthetic network to implement detoxification functionality. We employed a modular design strategy by engineering toxin-induced control of an enzyme scavenger. Our miRNA switch results show moderate synthetic expression control over a biologically active detoxification enzyme molecule, using an established design protocol. However, following a new design approach, we demonstrated an evolutionarily designed miRNA switch to more effectively activate enzyme activity than synthetically designed versions, allowing markedly improved extrinsic user-defined control with a toxin as inducer. Our straightforward new design approach is simple to implement and uses easily accessible web-based databases and prediction tools. The ability to exert control of toxicity demonstrates potential for modular detoxification systems that provide a pathway to new therapeutic and biocomputing applications.

## Introduction

Biocompatible information processing is critical for developing autonomous biological therapeutics or biosensors. Currently, biological computation can reprogram nucleic acids to store and process information^1–8^, modulate protein translation at transcriptional levels^9–13^, and perform biosensing functions^14–17^. However, designing molecular systems to regulate behaviour also requires programming high degrees of functionality into biochemical networks. Such advancement of synthetic gene expression control is critical to progress synthetic biology tools^18^. However, while the synthetic biology toolbox has made rapid progress using prokaryotes, the expansion of eukaryotic tools is still lacking. To date, only one type of riboswitch, thiamine pyrophosphate, has been identified for eukaryotes, in filamentous fungi and plants^19^ compared to the identification of around 40 families of riboswitches for prokaryotes^20^. At the eukaryotic transcriptional level, engineered promoter regulation has been reported^9,10,13^ but is challenging^21^, as it requires pairing of a protein surface with a DNA binding motif, and transport of activators and repressors into the nucleus. At the post-transcriptional level, engineering networks have focused on regulation of miRNAs^22–26^.

miRNAs regulate several physiological relevant biological processes, such as cell growth, differentiation, development and apoptosis, and miRNA disfunction is implicated in various diseases^27^. miRNAs thus make excellent candidates to design engineered switches as synthetic biology tools for gene regulation applicable in higher eukaryotes. Such engineered post-transcriptional control networks have been demonstrated using artificial miRNAs with embedded aptamers^23,26^, including engineered reversibility (7). Notably, drug-induced miRNA switches were used to demonstrate control of T cell signalling pathways, making progress towards the development of synthetic biology therapeutic tools for mammalian systems^25^. However, while miRNA-based gene expression control systems produce ON states close to maximal gene expression, OFF states show leakage^23^. This is a critical limitation when regulating enzyme turnover function, because only highly stringent switches will achieve the desired control, compared to the regulation of proteins without any catalytic function, such as previously published approaches controlling the fluorescent protein GFP^23^ and a cytokine receptor^25^.

In this study, we interrogated an engineered mammalian miRNA-based switch to tune the expression of an enzyme scavenger in the presence of a toxic small molecule, towards a futuristic vision of therapeutic or biocomputing implementation of synthetic tools with detoxification functionality. Cytochrome P450s play a substantial role in chemical toxicology. Many well-defined examples of roles of Cytochrome P450s exist in reducing adverse effects of toxins through biotransformation or bioactivation^28^. Toxicological extrapolations of animal test results to humans are problematic, not only because of variations in the metabolism, but also because of genetic variations in human Cytochrome P450s as understudied factor^29^. Cytochrome P450 1A2 (CYP1A2) was selected as the proof-of-concept detoxification enzyme, since Cytochrome P450s are known to metabolize approximately 60% of marketed pharmaceutical compounds^29,30^, and CYP1A2 metabolises several clinically important pharmaceuticals^31,32^ including 90% of theophylline in adults^33^. We paired CYP1A2 with detoxification of theophylline by integrating the high affinity and specificity theophylline aptamer^34^ in the basal stem of the miRNA. Results demonstrate improved potential for a reengineered mammalian miRNA-based system to control the expression of a biologically active detoxification enzyme molecule. Importantly, no previous study has focused on creating new detoxification tools based on conditional gene expression of an enzyme scavenger, and assessed its application potential for toxicological studies. Our results provide a new pathway for application of Cytochrome P450s towards personalized medicine, and support the development of miRNA switches as controlling elements for biocompatible information processing systems.

## Results

### User-defined control over intracellular molecular activation of an enzyme scavenger

**Figure 1:**
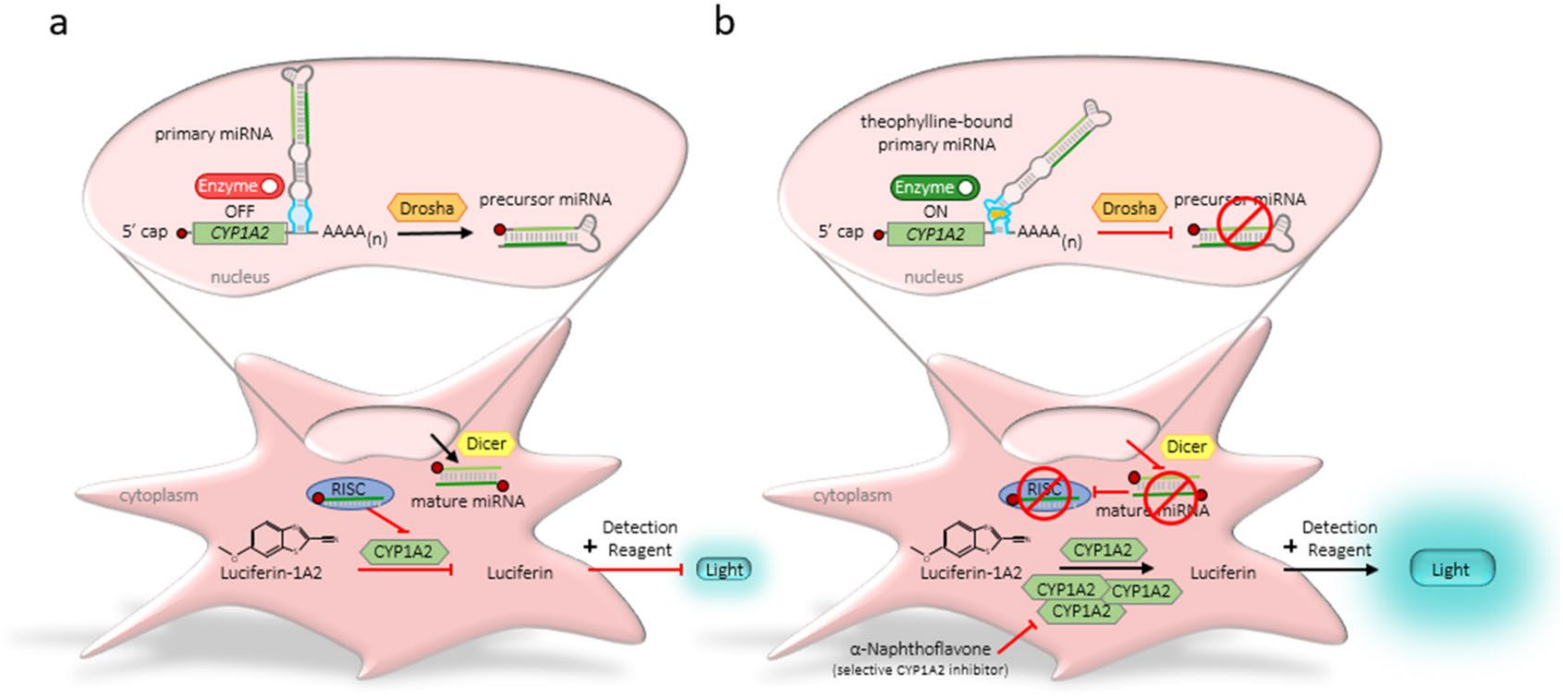
Scheme of CYP1A2 enzyme activation by theophylline using a theophylline-responsive miRNA switch. An RNA construct with a miRNA containing a theophylline-responsive structure-switching aptamer in the 3′ untranslated region (UTR) of CYP1A2 is depicted in the nucleus of a HEK-293 cell. While the exact mechanism of miRNA processing is unresolved, we assume the primary miRNA is processed by Drosha resulting in the precursor miRNA. Dicer cleavage produces a double-stranded mature miRNA in the cytoplasm^35,36^. One strand is incorporated into the RNA-induced silencing complex (RISC) and regulates CYP1A2 messenger RNA translation^36,37^. CYP1A2 enzymatic activity is indirectly measured by the detection of Luciferin, after conversion from externally supplied Luciferin-1A2 representing an easy read-out system. Luciferin-1A2 is converted by CYP1A2 into a luciferin precursor. The luciferin precursor is rapidly converted into luciferin following the addition of D-Cysteine, which is required for the reaction to occur and supplemented in the detection reagent. (**a**) In the absence of theophylline, CYP1A2 gene silencing occurs via RISC activation and CYP1A2-derived luminescence level is low. (**b**) In the presence of theophylline, the aptamer is bound. The secondary structure of the miRNA switch is changed, which masks the primary miRNA from processing^23^. CYP1A2 enzyme activity increases the luminescence levels. If α-naphthoflavone is added, it can selectively inhibit CYP1A2 activity, serving as a control. The mature miRNA sequence (guide strand) is shown in dark green and miRNA* (sense strand) in light green. The theophylline aptamer sequence is shown in light blue, and the corresponding bulge area highlighted by blue, theophylline is depicted in orange in the aptamer region of the theophylline-bound primary miRNA. The 5′ cap is displayed as red circle and AAAA(n) indicates the poly (A) signal of the mature messenger RNA. CYP1A2 enzyme translation status controlled by the miRNA switch is additionally depicted as ‘Enzyme OFF’ (red switch) and ‘Enzyme ON’ (green switch).

In this study, we investigated the ability of miRNA switches to turn on and tune the activation of an enzyme scavenger using a toxin, by harnessing the cells natural RNA interference system. Gene silencing was induced by an miRNA with a structure-switching aptamer embedded within its sequence^23^. If the aptamer was bound by the toxin, the secondary structure of the miRNA changed, masking the primary miRNA from processing, resulting in increased CYP1A2 expression. CYP1A2 enzyme scavenger activation was coupled to a luminescence read-out system to visualize changes (Fig. 1).

**Figure 2:**
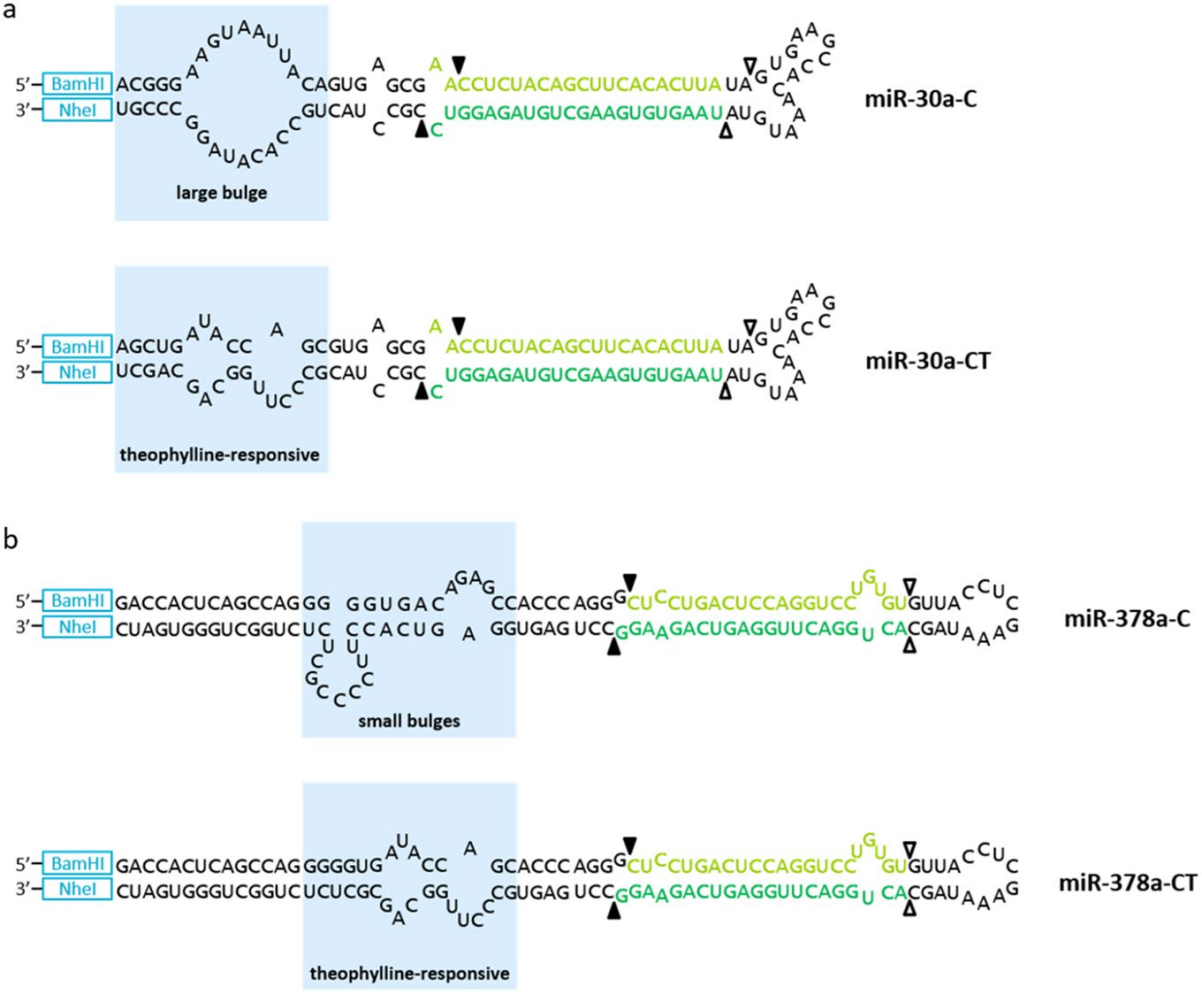
Secondary structure and sequence of primary miRNAs targeting CYP1A2 without (top) and with (bottom) a theophylline aptamer in the basal segment. Design based on (**a**) miR-30a targeting the CYP1A2 sequence starting at nucleotide 325 or (**b**) naturally occurring miR-378a. Bulge sequences are highlighted by blue. Black arrows indicate predicted Drosha cleavage sites and white arrows indicate predicted Dicer cleavage sites. Restrictions sites are shown in blue. The mature miRNA sequences (guide strands) are shown in dark green and miRNA* (sense strands) in light green.

We initially designed miRNA sequences to control CYP1A2 expression using free online tools, following general miRNA design rules^38,39^ and an established protocol^24^. We identified 5 sequences that target CYP1A2 (miR-30a-C), and from these designed theophylline-responsive primary miRNAs as switches (miR-30a-CT; Fig. 2a, Table 1). In addition, we designed a miRNA switch following a new design approach, which took advantage of a naturally occurring miRNA (Fig. 2b, Table 1). We obtained the sequence of the naturally occurring miR-378a^40^, including structural information and targeted region within the coding sequence of CYP1A2 (miR-378a-C), by using the two databases, miRbase^40^ and miRWalk Version 3^41^, respectively. Accordingly, we redesigned the miR-378a-C oligonucleotide sequence to encode for theophylline-dependent control by integrating the theophylline aptamer into the basal segment (miR-378a-CT). Plasmids expressing the above-described miRNA constructs, or CYP1A2 only as a control (no miRNA) (Fig. S1b,c), were constitutively co-expressed with cytochrome P450 oxidoreductase (POR). POR is known to supply electrons to Cytochrome P450 enzymes, which originate from NADH or NADPH and are required for proper catalytic function of the oxidation reactions. The effective co-transfection concentration of POR for optimal CYP1A2 activity in HEK293 cells was determined using the CYP1A2 only construct (Fig. S2).

**Table 1:**
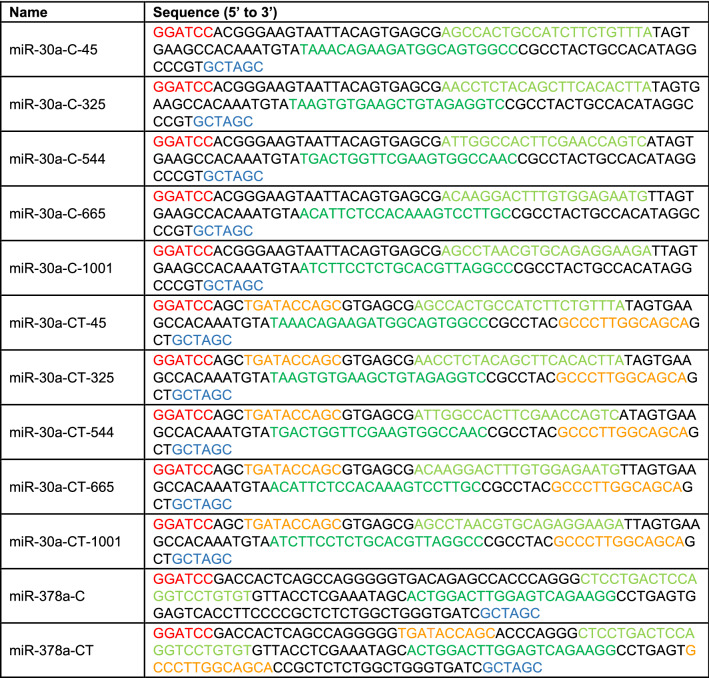
Sequences for control and theophylline-responsive primary miRNAs.

Primary miRNAs were cloned into BamHI/NheI within pSF-CMV-CYP1A2 downstream of the CYP1A2 coding region. Color codes: red, BamHI; blue, NheI; light green, sense strand sequence; dark green, guide strand sequence; orange, theophylline aptamer.

### miRNA silencing of CYP1A2 expression

**Figure 3:**
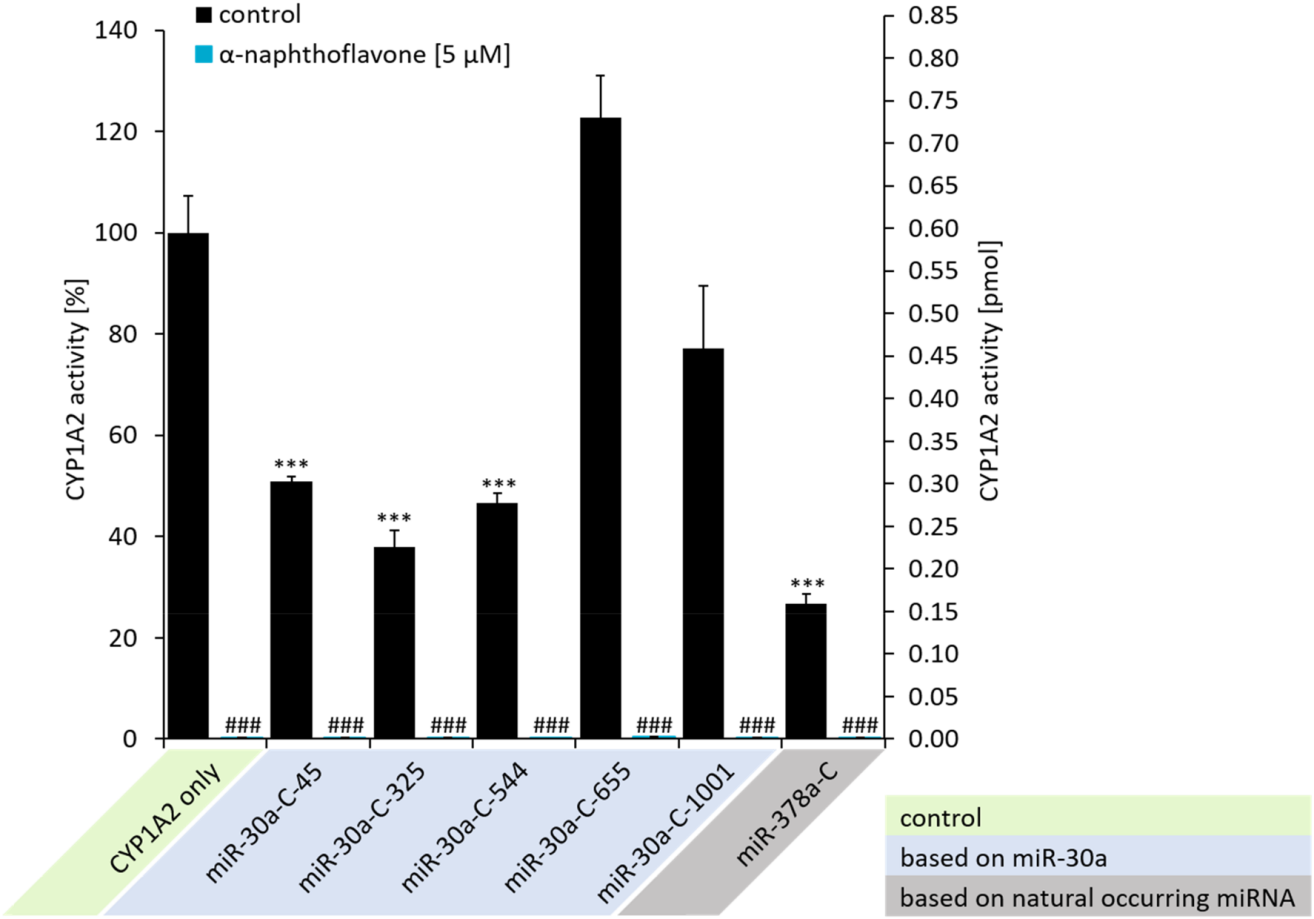
Demonstration of miRNA-mediated gene silencing in HEK-293 cells transfected with miRNA-C plasmid constructs, determined by luminescence assay measuring CYP1A2 enzyme activity. CYP1A2 activity (%)/(pmol) was measured in absence and presence of 5 µM α-naphthoflavone, a selective CYP1A2 inhibitor. CYP1A2 enzyme activity (pmol) was calculated with a calibration curve (Fig. S3) using recombinant CYP1A2 (mean ± standard deviation; n = 3; ***P < 0.001 versus no miRNA; ^###^P < 0.001 versus control).

After establishing optimal CYP1A2 expression for the CYP1A2 only construct, we then examined the gene silencing properties of our designed miRNA sequences, in the absence of the theophylline responsive switch (Fig. 3). Three out of five miRNA sequences designed using the previously established protocol (miR-30a-C-45, 325 & 544), significantly reduced the CYP1A2-derived luminescence activity to remaining activity levels ranging from 38 to 51%. However, two failed to induce significant gene silencing (miR-30a-C-665 & 1001) with remaining luminescence activity levels of 77% and 123%, suggesting that targeting CYP1A2 sequence at the 3′ end of the protein was more effective in attenuating messenger RNA translation. In contrast, our new design based on the naturally occurring miRNA (miR-378a-C) significantly reduced CYP1A2 enzyme activity to 27% residual luminescence activity, supporting the effectiveness of our new design approach, which reduced the need of designing multiple synthetic miRNAs compared to identifying one naturally occurring miRNA. Notably, treatment with a CYP1A2 selective inhibitor (α-naphthoflavone) excluded any background enzyme activity in HEK-293 cells (Fig. 3, residual activities ranged from 0.13 to 0.46%).

### Synthetic control of CYP1A2 enzyme production with theophylline-responsive miRNA switches

**Figure 4:**
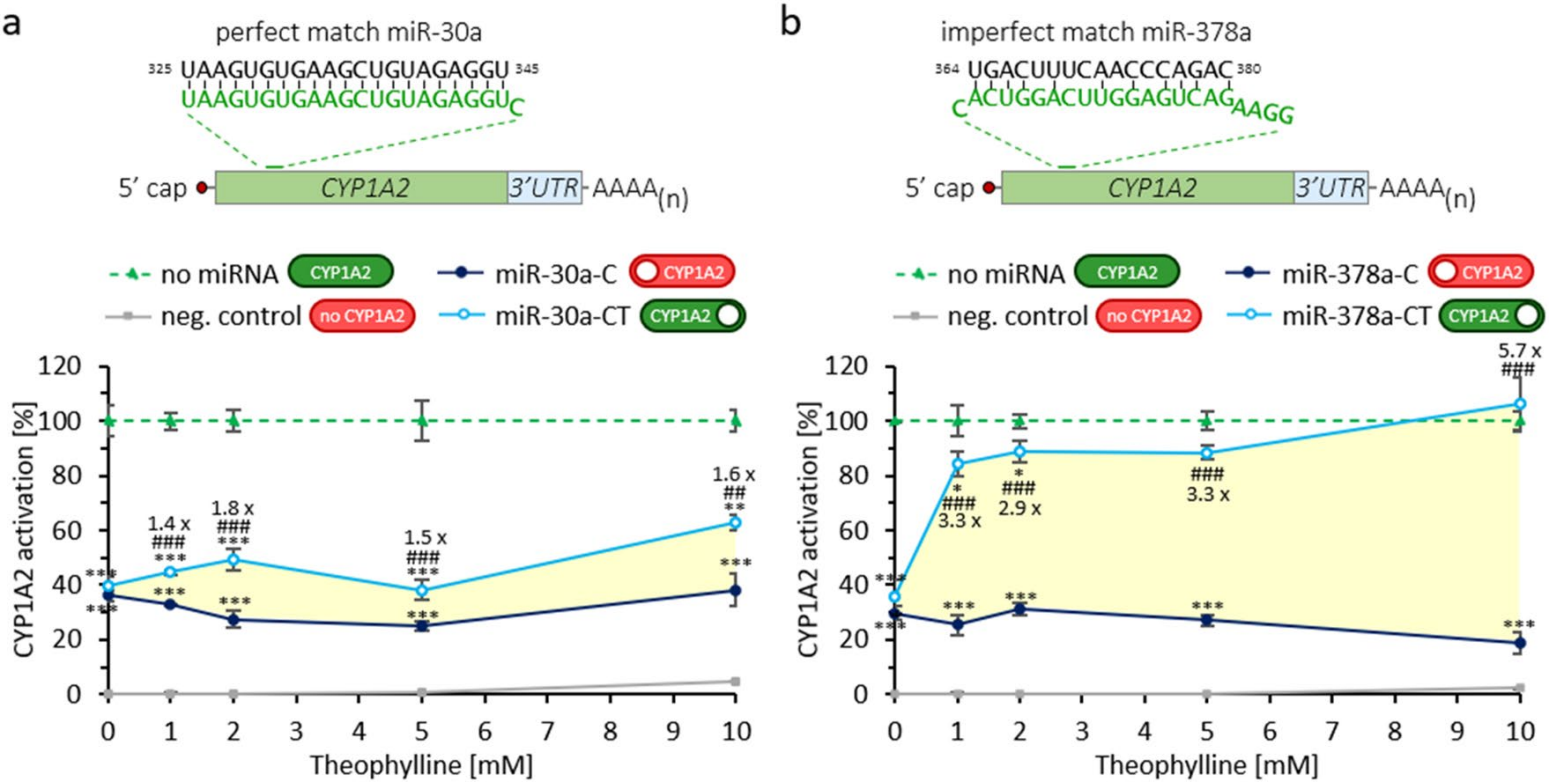
Increased CYP1A2 activation in HEK-293 cells transfected with miR-30a-CT (**a**) and miR-378a-CT (**b**) in the presence of theophylline compared to not theophylline-responsive miRNAs miR30a-C and miR378a-C, compared to no miRNA control (100%) at each concentration of theophylline (CYP1A2 enzyme only). Respective match binding site in CYP1A2 is depicted on top. Percent CYP1A2 activation (%) for each construct is shown as mean ± standard deviation (n = 3). Firefly luciferase is used as negative control (neg. control; no CYP1A2 enzyme). Legend: *P < 0.05, **P < 0.01, ***P < 0.001 miR-C and miR-CT compared to no miRNA; ^###^P < 0.001 miR-CT compared to miR-C). The expected CYP1A2 enzyme expression status for each construct is illustrated with switches: green corresponds to ‘ON’ and red depicts ‘OFF’, white circle depicts switching capability.

The two miRNA sequences that most effectively silenced CYP1A2 expression (miR-30a-C-325 and miR-378a-C) were then used to determine performance of designed theophylline-responsive miRNA switches (miR-30a-CT and miR-378a-CT). We assessed the user-defined regulation over intracellular enzyme scavenger activity with varying concentrations of theophylline (0–10 mM). As anticipated only the miRNA switches with an integrated theophylline aptamer sequence (miRNA-CT) responded to the presence of theophylline, inducing CYP1A2 enzyme activity measured with a luminescence assay (Fig. 4). Without addition of theophylline, as expected, both the control (miR-30a-C) and theophylline-responsive (miR-30a-CT) miRNAs resulted in low CYP1A2 enzyme activities compared to CYP1A2 only (Fig. 4a; residual activities 36% and 40%). In presence of theophylline, we observed small but significant fold-increases in CYP1A2 enzyme activity, of up to 1.8-fold, in the miRNA containing the theophylline aptamer (miR-30a-CT) compared to the miRNA without the theophylline aptamer (miR-30a-C) (Fig. 4a, 27% to 49% at 2 mM theophylline). Similarly, in the naturally occurring miRNAs without (miR-378a-C) and with (miR378a-CT) the theophylline switch, in the absence of theophylline, a similar reduction of CYP1A2 enzyme activity was observed compared to CYP1A2 only (Fig. 4b; residual activities 30% and 36%). However, our naturally occurring miRNA with the theophylline aptamer (miRNA-378a-CT) induced increases of CYP1A2 enzyme activity of up to 5.7-fold compared to the miRNA without the theophylline aptamer (miRNA-378a-T) (Fig. 4b, 84% at 1 mM theophylline, 106% at 10 mM theophylline). The naturally occurring miRNA thus demonstrated markedly improved user-defined control over the intracellular molecular activation of CYP1A2 enzyme activity, supporting our new design approach, and achieving close to maximum enzyme expression levels in presence of toxin. Note, a theophylline concentration-dependent decrease in luminescence signals in the CYP1A2 only control was observed (Fig. S4a 5% and Fig. S4b 8% activity at 10 mM theophylline), suggesting a concentration-dependent cytotoxic effect of theophylline.

### Time-dependent control of CYP1A2 activity of the theophylline-responsive miRNA switch

**Figure 5:**
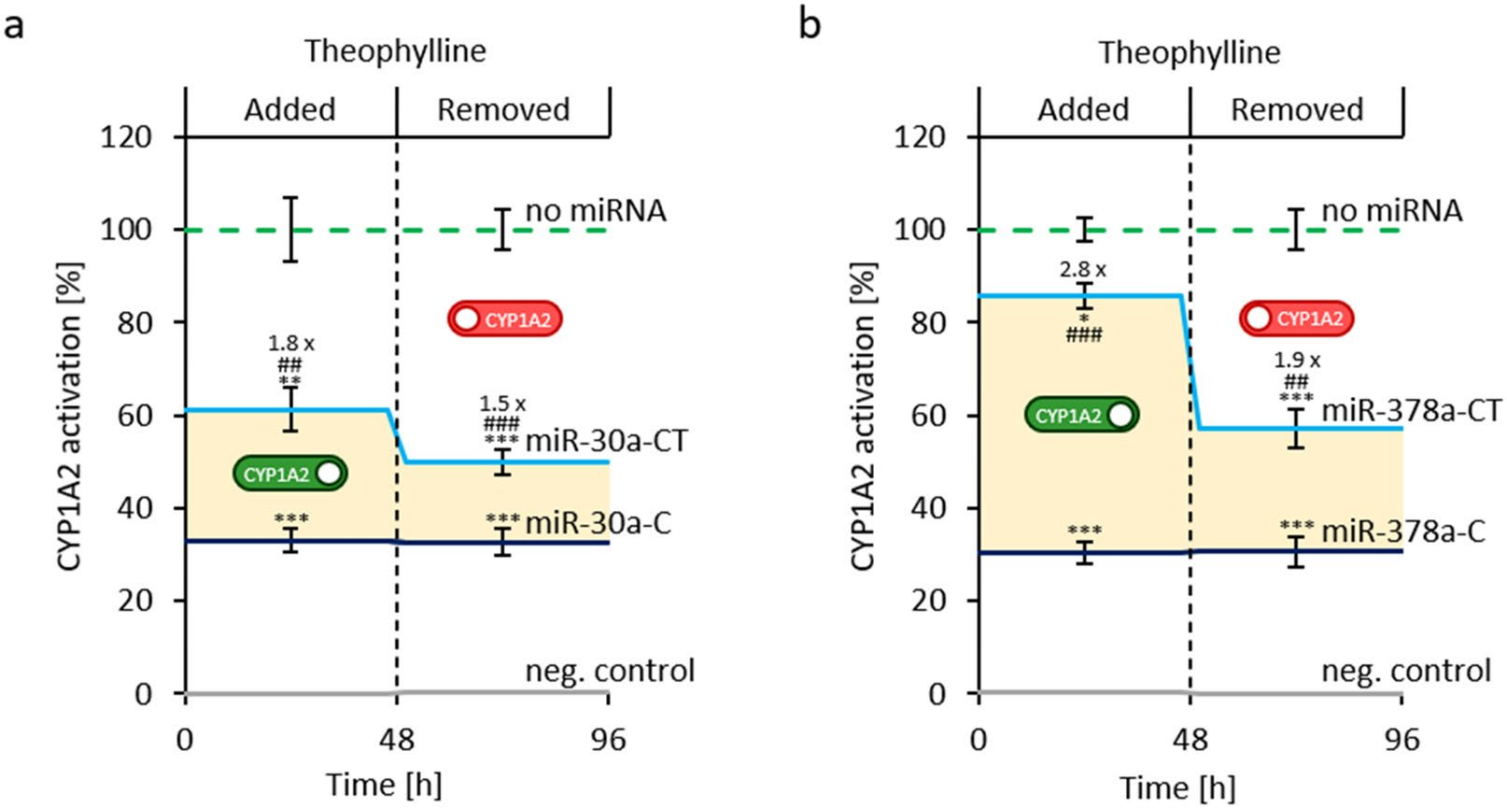
Time-dependent reversible CYP1A2 activation in HEK-293 cells transfected with (**a**) miR-30a-CT, and (**b**) miR-378a-CT measured after 48 h in presence of theophylline [2 mM] and after 96 h, which treated HEK293 cells for 48 h with theophylline [2 mM] and then removed theophylline for 48 h. Percent CYP1A2 activation (%) for each construct is shown as mean ± standard deviation (n = 3). No miRNA control (CYP1A2 enzyme) is set to 100%. Firefly luciferase is used as negative control (neg. control; no CYP1A2 enzyme). Legend: *P < 0.05, **P < 0.01, ***P < 0.001 miR-C and miR-CT compared to no miRNA; ^##^P < 0.01, ^###^P < 0.001 miR-CT compared to miR-C). The expected CYP1A2 enzyme expression status for the theophylline-responsive miRNA switch is illustrated: green depicts ‘ON’ and red corresponds to ‘OFF’.

After establishing the activation of CYP1A2 activity with our theophylline-responsive miRNA switches, we then investigated the dynamic properties of our switches by incubating in the presence of 2 mM theophylline for 2 days to turn on the theophylline-responsive miRNA switches, followed by removal of theophylline for another 2 days to turn off the miRNA switches. Compared to no miRNA control (CYP1A2 only; 100%), in the presence of theophylline, we observed the expected fold-changes in our miRNA switches (Fig. 5a 1.8-fold in miR-30a-CT, 33% to 61%; and Fig. 5b 2.9-fold in miR-378a-CT, 30% to 86%). In addition, removal of theophylline for 2 days resulted in a decrease in CYP1A2 activation (Fig. 5a miR-30a-CT 50%, miR-30aC 33%; and Fig. 5b miR-378a-CT 57%, miR-378a-C 31%), demonstrating our miRNA switches are reversible, with the greater reduction of activity (− 29% to 1.9-fold) observed for the naturally occurring miRNA (miR-378-CT) compared to the synthetically engineered miR-30a-CT (− 11% to 1.5-fold). As expected, no changes in enzyme expression was observed in our control miRNA switches that did not contain the theophylline aptamers (miR-30a-C and miR378-C), which continued to express low level CYP1A2 enzyme (33% and 30% respectively) throughout the addition and subsequent removal of theophylline. We note that the half-life of CYP1A2 is approximately 36 h^42^; while further reversibility may theoretically be observed over a longer period, longer monitoring was not possible in our transient expression system. Future studies may investigate autoregulatory feedback in more detail using stably transfected cells.

## Discussion

In this study, we successfully demonstrated that we can exert regulatory control on enzyme scavenger expression using a miRNA-based system, which is toxin-responsive. Our study expands on previous studies investigating synthetic RNA-based systems to construct logic circuits^7,8,11,12,14,15^ and miRNA-mediated gene expression control^23,26^. Engineering signal-responsive RNA circuits and switches has gained significant interest recently following the successful reprogramming of translation in yeast exploiting a mechanism termed − 1 programmed ribosomal frameshifting (− 1 PRF)^43^, which resulted in advances in synthetic RNA-based logic computation in HeLa cells^7,44^. Previously, aptamer-based biosensors have been coupled to enzymes to regulate gene expression^11,12^, but implementation in mammalian cells has been limited compared to applications of riboswitch-based sensors in prokaryotes^45–49^. Our results demonstrate that post-transcriptional gene regulation by miRNA switches can encode a detoxification function, which is dependent on an intracellular biosensing event. Such a detoxification functionality coupled to biosensing may have a wide range of applications in the future, such as in personalized medicine protecting humans from adverse toxins or in environmental monitoring and defence by engineering resilience to contamination.

Molecular behavior control is a critical tool for biocomputing^3,7–10,13,17,50,51^, and miRNA-induced translational control has emerged as a powerful system for exerting control in eukaryotes^44^. The advantage of programming cellular function with an RNA-based system to exert translational control lies within rapid protein function and fast response times to external signals. Beisel and colleagues pioneered ligand-responsive miRNAs, demonstrating twofold-change in GFP reporter expression^23^. Interestingly, miRNA clusters are separated by short spacers^52^ and, in the GFP study, miRNA expression levels were demonstrated dependent on spacer length^23^, improving GFP expression to 4.7-fold, albeit with a reduction in total GFP expression levels^23^, which reduced the considerable leakage in the OFF state, but did not eliminate GFP expression. In our study, we chose to incorporate only one copy in our engineered miRNA switches, to achieve close to maximum enzyme expression levels in presence of the toxin. In addition, secondary structures of terminal stem-loops in miRNAs are well known to be important sites for processing regulation^53^. We opted for the insertion of the aptamer at the basal segment of miRNAs to avoid changing the highly conserved terminal loop region, similar to previously published successful design approaches^23–25^. Small molecule addition, in our case theophylline, then ablates miRNA processing. This is presumably due to a structural change preventing processing by Drosha and Dicer, which prevents presentation of a mature miRNA to the RNA-induced silencing complex (RISC) (Fig. 1b).

One problem with miRNA-based gene expression control systems is they are capable of producing ON states close to maximal gene expression, whereas OFF states show rather high leakage. Despite mentioned limitations, tuneable control over T cell proliferation has recently been demonstrated with a drug-responsive miRNA, which targeted the subunit ß of interleukin-2 receptor, a cytokine receptor displayed on the surface of T cells^25^. Moreover, miRNA switches have been shown to control the gene expression of the HIV-1 entry inhibitor CD4-Ig, when the transgene was introduced using adeno-associated virus vectors in HeLa cells^44^. These studies demonstrate recent progress made towards the development of mammalian synthetic biology therapeutic tools for applications in health and medicine. Our toxin-induced regulatory system similarly pushes the boundaries towards developing miRNA switches for use in toxicological studies, but targets an enzyme with turnover that requires more stringent control to observe significant effects than proteins without any catalytic activity. Critically, we demonstrated the naturally occurring CYP1A2-targeting miR-378a^54^ showed the strongest silencing effect compared to designs made according to the established protocols. This construct also operated with the least leakage and most improved synthetic control at the lowest toxin levels. The study was intended to be a proof-of-concept for optimizing translational control of enzymes using miRNA switches, however, it is interesting to note that the therapeutic half-life of theophylline is more than a day in patients with impaired liver function^55,56^. An artificially embedded theophylline-responsive switch could assist with improving clearance. The small quantities of CYP1A2 produced in the inactive leak state of the switch could bolster the natural CYP1A2 liver enzyme activity (for example, by engineering non-liver cells to additionally produce CYP1A2). Then, if the patient was exposed to toxic levels of theophylline [> 100 μM^57^], such as during emergency asthma treatment, the embedded switch would become activated to rapidly detoxify and improve the half-life of theophylline clearance.

Our results confirmed our intuition that the evolutionarily-designed miRNA would be more effective in enzyme activation in the presence of a toxin inducer than the synthetically designed versions, improving the user-defined extrinsic control to turn on and tune enzyme activity. Importantly, control exerted by theophylline-responsive miRNA switches is reversible, indicated by a measured decrease of CYP1A2 activity after removal of theophylline. This straightforward new design approach is simple to implement as it involves searching for naturally occurring miRNAs^40^, and free web-based databases and prediction tools^41,58^. The miRNA we chose is highly conserved among mammalian species including human, mouse and rat^40^, and should therefore work in all mammalian cells. In general, miRbase has stored miRNAs targeting 271 species, including animals and plants^40^. Our system would be expected to work in any species that harbor the necessary biogenesis components for miRNA processing, such as the RNase III enzymes Drosha and Dicer^35,36^. The miRNA-378a may have shown improved control due to its use of imperfect hybrids within the miRNA structure, which has been shown to allow individual miRNAs species to interact with multiple targets^35^. Perfect hybrid formation has the advantage of achieving better target prediction and reliability in predicting off-target RNA interference activity^59^, but may impede with essential miRNA features^37^. The limitation of this new design approach is its dependence on naturally occurring miRNAs which may not show the required level of gene silencing to start with. Leakage in the OFF state may be addressed by incorporating multiple miRNA copies, likely trading tighter control for a reduction in maximum toxin-induced enzyme activity, as previously shown^23^. However, leakage also may be favourable for futuristic protective tools decreasing adverse effects of drugs and toxins in patients, providing baseline protection that can markedly increase if a corresponding toxin is detected by the system. Our easily adaptable system not only expands the mammalian synthetic biology toolbox, but could also be useful in the elucidation of biological roles for small non-coding RNAs and its targets. Further, our system could be used in toxicity screening applications, enabling assessment of the impact of genetic variations on the biotransformation and bioactivation of toxins and drugs, paving the way towards personalized medicine. Future studies may investigate the delivery of our miRNA switch approach in an animal model by encoding the miRNA/aptamer/gene cassette into plasmid DNA^60^, or a viral vector^61–63^, and delivering to cells either systemically (intravenous or intraperitoneal) or locally (e.g. intratumoral injection) via standard delivery systems. In vivo delivery systems successfully demonstrated in animal models include cationic polymer polyethylenimine^64^, inorganic nanoparticles^65,66^, cationic lipids and liposomes^67,68^, and atelocollagen^69^.

Despite endeavours to translationally control mammalian gene regulatory processes, this does not come without its obstacles. Most of the recent approaches use an aptamer element to engineer ligand-responsiveness and to regulate gene expression^23–26,44^. Aptamers are widely used, due to their high binding affinity against their respective target and their specificity^70–82^. In principal, the process of SELEX (Systematic Evolution of Ligands by Exponential enrichment) allows aptamer selection against any small molecule of interest^83,84^. However, well-characterized RNA aptamers for small organic molecules, especially with desired high binding affinity, are low in availability^34,85–90^ opposed to DNA aptamers^91–107^ and this represents a major limitation in context of multiplexing. To engineer modularity and achieve multi-input with multi-output systems, use of different aptamers is critical and, in the future, will allow programming of functions running in parallel resulting in more sophisticated logic-based circuits. Our data suggests this can be achieved by embedding known or de novo aptamers that bind the desired input molecule within the basal segment of the miRNA, and the most optimal miRNA sequences are likely to be found by searching miRNA databases targeting the gene of interest. Further, one can envision that interchangeable, modular tools could combine both a visual output (reporter gene expression, e.g. fluorescence or bioluminescence) and any chemical reaction (catalyzed by an enzyme, e.g. toxin break-down). This would enable the use of such tools in a wide variety of processes beyond health and medicine.

## Conclusion

In this study we successfully demonstrated user-defined control over intracellular molecular activation of an enzyme scavenger by using miRNA switches with an embedded aptamer sequence to synthetically control enzyme production. Our proof-of-concept system shows how post-transcriptional gene regulation by miRNA switches can encode a detoxification function, indicating potential for application in protecting humans from toxic molecules, and provides an example for molecular behavior control. We note that efficient control of mammalian gene regulatory processes on translational level, needs to address a major obstacle, the limited number of available and suitable RNA aptamers with proper characterization, which significantly impedes with engineering desired modularity. However, high interest in synthetic gene expression control supports efforts made to engineer miRNA switches as smart controlling elements for biocompatible information processing systems. Successful developments will be essential for future implementation of autonomous biological therapeutics or coupled smart biosensors able to exert detoxification function.

## Methods

### Constructing plasmids with miRNA switches

All oligonucleotides were synthesized by GenScript (New Jersey, USA or Nanjing, China) and resuspended in ultra-pure nuclease-free water (Astral Scientific, NSW, Australia). The coding region of mouse cytochrome P450, family 1, subfamily a, polypeptide 2 (CYP1A2; NM_009993.3) and mouse cytochrome P450 oxidoreductase (mPOR; NM_008898.2) were synthesized with the consensus Kozak sequence (GCCACC) and cloned into the EcoRI/ClaI restriction sites of pSF-CMV-FLuc (Oxford Genetics Ltd, Oxford United Kingdom) expression vector to remove the Firefly luciferase coding region and create pSF-CMV-CYP1A2 and pSF-CMV-POR (Fig. S1a,b), respectively.

Based on miR-30a^24^, control and ligand-responsive miRNAs reported in Table 1 were cloned into BamHI/NheI downstream of the CYP1A2 coding region into pSF-CMV-CYP1A2 to create various versions of pSF-CMV-CYP1A2-miR (Fig. S1c). MiRNAs targeting CYP1A2 were designed by either adhering to previously published design rules^38,39^ [position 45 and 325] or using online tools from InvivoGen (https://www.invivogen.com/sirnawizard/) [position 544] and Dharmacon (https://horizondiscovery.com/en/products/tools/siDESIGN-Center) [position 665 and 1001] (see Table 1). We used the miRbase database (http://mirbase.org/) to obtain the sequence of the natural occurring mmu-miR-378a^40^ and the miRWalk Version 3 database (http://mirwalk.umm.uni-heidelberg.de/) that provides in silico meta-analysis by combining data sets of 13 different prediction algorithms^41^, to predict a region in the coding sequence (CDS) of mCYP1A2, which is targeted by mmu-miR-378a. The natural occurring miRNA mmu-miR-378a was predicted to target mCYP1A2 within its coding sequence at 2 positions (364–380 and 1552–1579). We chose to verify the algorithm-predicted miRNA binding site, which lays in closer proximity of the start codon. Finally, we analyzed the oligonucleotide sequence with mfold (http://unafold.rna.albany.edu/?q=mfold)^58^, a nucleic acid folding and hybridization prediction web tool, followed by the redesign of mmu-miR-378a for user-defined ligand-dependent control.

All constructs were sequence-verified (GenScript, New Jersey, USA or Nanjing, China). All restriction enzymes were purchased from New England Biolabs.

### Preparing expression plasmids

One Shot MAX Efficiency DH5α-T1^R^ competent cells (Invitrogen, Massachusetts, USA) were incubated with 1 µl of plasmid on ice for 30 min. These cells were then heat shocked at 42 °C for 30 s, returned to ice for 2 min, and grown in 950 µl SOC media (Invitrogen, Massachusetts, USA) at 37 °C with shaking for 1 h. Resulting transformed *Escherichia coli* cells were plated on LB agar with ampicillin (100 µg/ml; Sigma Aldrich, Missouri, USA) and grown over night at 37 °C. Colonies were then grown 8 h in 10 ml LB medium with ampicillin (100 µg/ml) followed by growing 400 ml to extract plasmids. Transfection-grade plasmid DNA was obtained using the NucleoBond Xtra Midi or Maxi kits (Macherey–Nagel, Düren, Germany). Final DNA product was assessed for quantity and quality using NanoDrop 200- (Thermo Fisher Scientific, Massachusetts, USA).

### Expressing enzymes and miRNA switches

HEK 293 cells were grown in high glucose Dulbecco’s Modified Eagle Medium (GIBCO, Thermo Fisher Scientific, Massachusetts, USA) supplemented with 10% fetal calf serum, 100 µg/ml streptomycin, and 100 IU/ml penicillin at 37 °C with 5% CO^2^ and 95% humidity. Cells were transiently transfected with respective plasmids using Lipofectamine LTX Reagent (Invitrogen, Massachusetts, USA) according to the manufacturer’s instructions 1 day after seeding. On the day of transfection, the media was supplemented with theophylline (Sigma Aldrich, Missouri, USA) at specified concentrations. Theophylline concentrations were selected to maximize the regulatory response without severely compromising cell viability over the course of the transient assays. For characterization of theophylline-responsive miRNA mediated CYP1A2 gene expression, co-expressions of plasmids were performed using a 1 to 1 ratio of POR DNA plasmid and corresponding CYP1A2 DNA plasmid, after identifying the 1 to 1 ratio as being optimal (Fig. S2).

### Measuring miRNA switch-induced control by luminescence

The luminescent P450-Glo CYP1A2 Induction/Inhibition assay (Promega, Wisconsin, United States) was used according to the manufacturer’s instructions to measure CYP1A2 enzyme activity, which was controlled by the control- or ligand-responsive miRNAs. The assays were performed 2 days post-transfection, except for the experiment which measured the time-dependent control of CYP1A2 activity, which performed additional assays 4 days-post-transfection. In short, cells were washed twice with HBSS containing salicylamide (3 mM, Sigma Aldrich, Missouri, USA), followed by a 1-h incubation with the Luciferin-1A2 substrate. The CYP1A2-specific inhibitor α-naphthoflavone (5 mM, Sigma Aldrich Missouri, USA) was used to ensure activities are CYP1A2-specific. Luciferin-1A2 substrate from incubated wells was diluted with the Luciferin detection reagent (1:1), incubated for 20 min and subsequently measured on the EnSight multimode plate reader (Perkin Elmer, Massachusetts, USA) with the integration time set to 1 s/well. Luminescence levels [%] were calculated after subtracting background activity levels from cell-free control wells. The positive control, samples transfected with CYP1A2 and POR (1:1), were set to 100%. Recombinant human CYP1A2 (Abcam, Cambridge, United Kingdom) was used to establish a calibration curve with the P450-Glo CYP1A2 luminescence assay (Promega, Wisconsin, United States). Data are presented as mean ± SD of 3 replicates of independently transfected wells in the same cell-culture plate. Statistical significance was determined by the Student’s unpaired two-tailed t-test. Group differences were considered significant for *P < 0.05, **P < 0.01, and ***P < 0.001. Experiments were repeated at least three times.

## Supplementary Information

Supplementary Information.
