# Supplementary material for: Translational control of enzyme scavenger expression with toxin-induced micro RNA switches
Source: Sci Rep. 2021 Jan 28;11:2462. doi: 10.1038/s41598-021-81679-6 (PMC7844233; doi:10.1038/s41598-021-81679-6)
Supplement: Supplementary file 1 — Supplementary Information. [file 41598_2021_81679_MOESM1_ESM.pdf]

SUPPLEMENTARY INFORMATION

Translational control of enzyme scavenger expression with toxin-induced micro RNA switches

Nina M. Pollak, [1,2,3,*] Justin J. Cooper-White[2,4], Joanne Macdonald[1,5,*]

[1]Genecology Research Centre, School of Science and Engineering, University of the Sunshine Coast, Sippy Downs, QLD, Australia

[2]Australian Institute for Bioengineering and Nanotechnology, The University of Queensland, Brisbane, QLD, Australia

[3]CSIRO Synthetic Biology Future Science Platform, GPO Box 1700, Canberra, ACT, Australia

[4]UQ Centre for Stem Cell Ageing and Regenerative Engineering, The University of Queensland, Brisbane, QLD, Australia

[5]Division of Experimental Therapeutics, Department of Medicine, Columbia University, New York, NY, United States

Table of contents

Figure S1: Plasmid maps.

Figure S2: Optimization of CYP1A2 enzyme activity by co-expression of POR, the principal redox partner of Cytochrome P450.

Figure S3: CYP1A2 luminescence assay calibration curve with recombinant human CYP1A2.

Figure S4: Increased CYP1A2 expression in HEK-293 cells transfected with miRNA switches in presence of varying concentrations of theophylline.

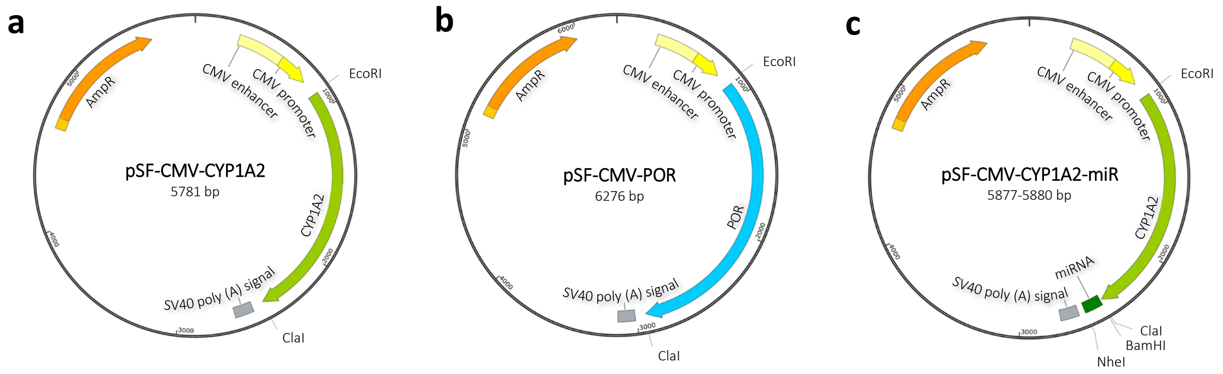

Figure S1: Plasmid maps. To obtain (**a**) pSF-CMV-CYP1A2 and (**b**) pSF-CMV-POR the mouse gene CYP1A2 or POR, respectively, were cloned into the EcoRI/ClaI restriction sites of pSF-CMV-FLuc with a consensus Kozak sequence (GCCCACC) immediately upstream of the start codon. CYP1A2 or POR are constitutively expressed from the mammalian Cytomegalovirus (CMV) immediate early promoter, which has the CMV enhancer upstream. (**c**) To generate the 12 versions of pSF-CMV-CYP1A2-miR, six miRNAs with and six miRNAs without theophylline aptamer in the basal segment of the primary miRNA sequence were cloned into BamHI/NheI restriction sites located in the 3' untranslated region of pSF-CMV-CYP1A2 and upstream of the SV40 poly (A) signal. Plasmid maps were produced using SnapGene software (GSL Biotech; available at https://www.snapgene.com). Abbreviations: AmpR, ampicillin resistance; CMV, Cytomegalovirus; CYP1A2, Cytochrome P450 1A2; POR, cytochrome P450 oxidoreductase; miR or miRNA, micro RNA.

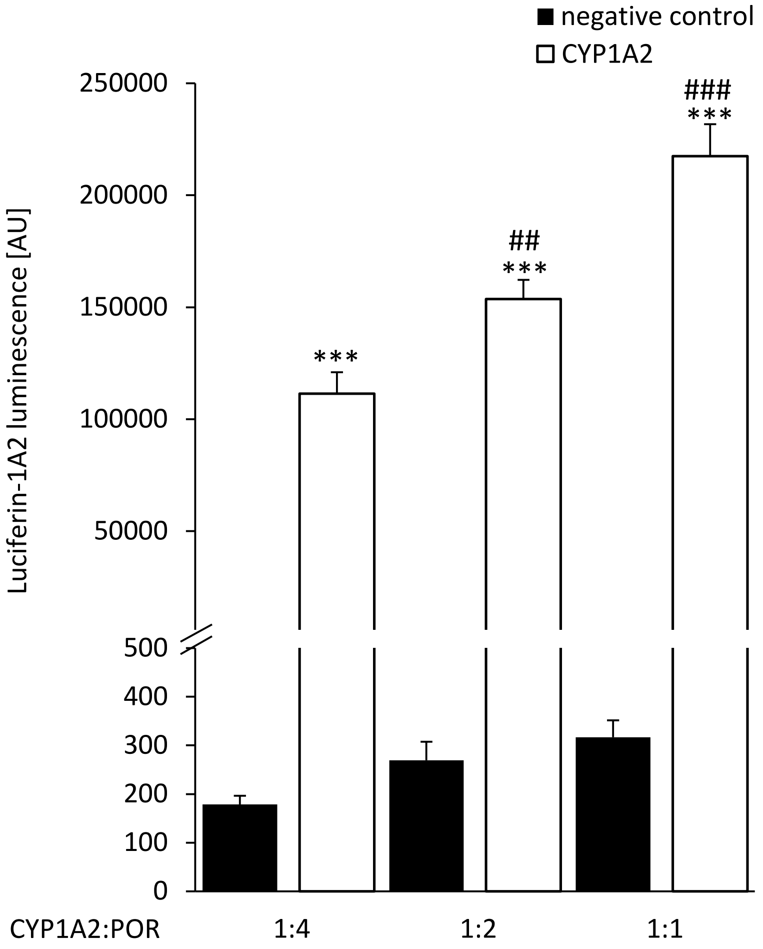

Figure S2: Optimization of CYP1A2 enzyme activity by co-expression of POR, the principal redox partner of Cytochrome P450. Luciferin-1A2 luminescence levels were determined at different ratios of CYP1A2 to POR, changing the POR amount. Corresponding wells were co-transfected with the fixed amount of 100 ng CYP1A2, which was mixed with either 100 ng POR + 300 ng negative control, 200 ng POR + 200 ng negative control or 400 ng POR. Luminescence was measured 72-hours post transient transfection with indicated constructs (n = 3; *** P < 0.001 versus negative control; ## P < 0.01 and ### P < 0.001 versus same CYP1A2:POR ratio). Abbreviations: AU, arbitrary units; CYP1A2, Cytochrome P450 1A2; POR, P450 oxidoreductase.

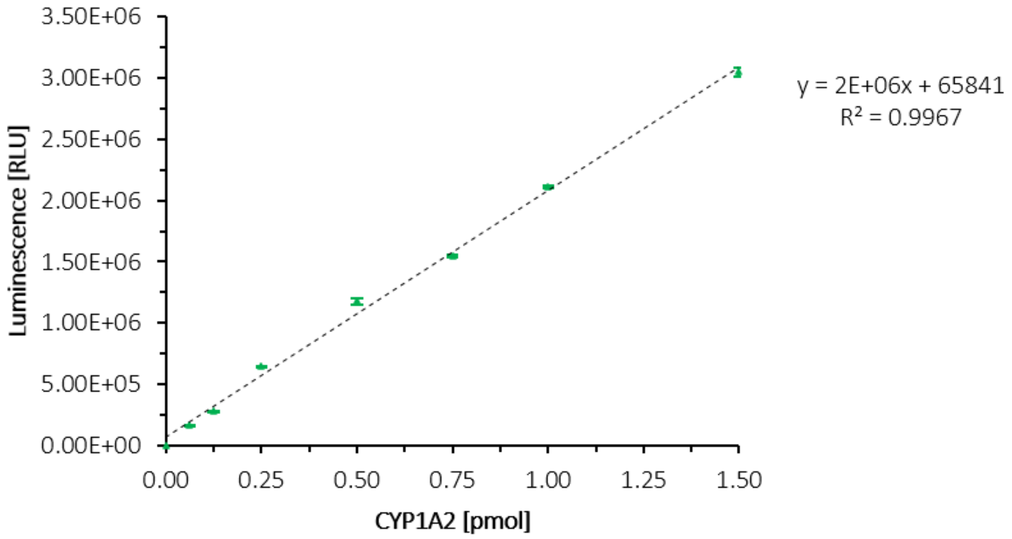

Figure S3: CYP1A2 luminescence assay calibration curve with recombinant human CYP1A2. P450-Glo CYP1A2 Assay (Promega, Wisconsin, United States) was performed with a range of CYP1A2 concentrations. Substrate concentration (6 µM Luciferin-1A2) and incubation time (10 min, 37°C) were as recommended by the manufacturer. Recombinant human CYP1A2 was purchased (Abcam, Cambridge, United Kingdom).

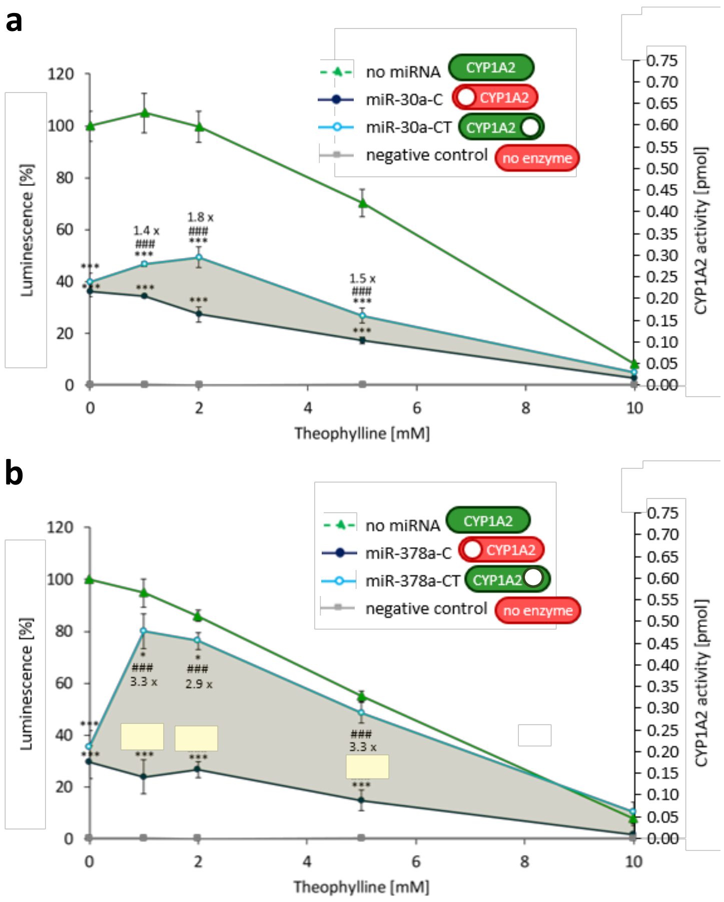

Figure S4: Increased CYP1A2 expression in HEK-293 cells transfected with miR-30a-CT (**a**) and miR-378a-CT (**b**) in presence of theophylline, compared to not theophylline-responsive miRNAs (miR30a-C and miR378a-C), and compared to no miRNA control, at varying concentrations of theophylline. Percent luminescence (%) and CYP1A2 activity (pmol) for each construct is shown as mean ± standard deviation (n = 3). CYP1A2 enzyme activity (pmol) was calculated using a calibration curve (Figure S3) using recombinant CYP1A2. Firefly luciferase is used as negative control (no CYP1A2 enzyme). Legend: * P < 0.05, *** P < 0.001 miR-C and miR-CT compared to no miRNA; ### P < 0.001 miR-CT compared to miR-C). The expected CYP1A2 enzyme expression status for each construct is illustrated with switches: green corresponds to ‘ON’, red depicts ‘OFF’ and white circle depicts switching capability.
